# Modern Sociology

**Published:** 1899-03-04

**Authors:** 


### Modern Sociology.

### THE COMPETITION OF THE PAUPER.

The periodic outcry against the competition of prison-made goods in our markets might have a sequel which would be perhaps more reasonable and to the point. Why is there no complaint against the competition of the pauper? Not the foreign pauper, at least not while he remains foreign, nor even the "destitute alien" of whom we have heard so much; but the native-bred pauper of our slums. The gist of the complaint against prison-made goods is that they are, as a matter of fact, sold at a loss. That is, that a Government, considering that it is bound to feed, clothe, and in every way provide for its criminals in any case, regards all it makes by the sale of the articles they produce during their incarceration as so much to the good, and does not carefully cast up, as the private employer does, the total cost of the production of these. It has been shown that prison labour, being forced labour, from which the worker considers that he gets no benefit, is not as energetic as free labour would be, and, therefore, if the free man and the prisoner competed on ordinary terms there would be little market for the latter's goods. It would be proved that they took longer to make, that in all probability they were not so good, and that, if you regarded as part of the cost of production not only the worker's food and clothing, but his proportionate share of the up-keep of the prison, and the expense of the very elaborate and constant supervision he requires; in short, if you looked on him as you do on the ordinary worker, and all the expense he costs his ungrateful country as his wages, it would be obvious that if a net profit were to be made on the results of his industry the things he makes would have to be sold at a much dearer rate than the productions of the free worker. But as a matter of fact his employer, the State whose laws he has broken, does not analyse his work in that way. All that we have called the prisoner's wages the State regards as inevitable expenses; and, selling prison-made goods for any price they will fetch, looks upon the price obtained as profit. It is thus that competition between these goods and those made by free labour becomes unfair.

Exactly the same reasoning applies to pauper labour. If it be thought that the pauper has, in any case, to be clothed and fed, and that anything, however little, that he can be made to earn is so much to the good, there is the same danger that his work will be used to compete with free labour. In the management of our workhouses we have nothing to complain of. The labour of the inmates is employed only in doing work within its walls, for the most part work of a desultory character, the wages of which would be difficult to estimate at market rates, and in any case this labour does not compete in the open market. But the case is very different when it is that of a person receiving outdoor relief.

Those to whom the guardians are most willing to give outdoor relief are, as a rule, the best of the pauper class, persons who have a certain self-respect, and are willing to work to the extent of their powers, if by this means they can keep a home of their own and avoid that dread ultimatum, "the house." They do not ask the guardians to maintain them wholly, but are content with a little "grant in aid." Now, therein lies the trouble. The guardians give the applicant half-a-crown a-week, and feel generous. The recipient accepts it, and, perhaps, feels grateful, and goes away saying that "with that help" he or she—it is generally a woman—can keep herself. This means that she will take work that takes up all her time, and for this will accept a wage which cannot by any means wholly maintain her. It takes both sources of income, the work and the dole, to make a living.

From the moral point of view we do not condemn the poor, hard-working creature, who does not desire to be wholly dependent upon charity; but from the economic point of view we are bound to inquire how her labour affects the woman who has no subsidy from the parish to aid her. Wages, unless kept up by the small number of the workers, or by their united determination to accept nothing less than a fixed wage, tend, like most other things in nature, to find their lowest level. The lowest level is always that of the subsidised pauper worker, whose demands thus become the measure of the rest. There is no doubt that the low wages ruling in many trades, especially the underpaid needlework done by women, are due in part to the competition of workers who have some other source of income than their own labour. With some it is merely a bywork, the woman's trifling contribution to the family income, and sometimes, as we have said, it helps to make up to a sufficient income with the parish dole of outrelief. Perhaps the dole is not given by the parish, but by the church or some charitable society; but in any case the result is the same. The work is done for, literally, starvation wages—wages on which no human being could live for the time it takes to do it.

For, be it noted, even with the aid given by charity, it is all but starvation. The worker with the trumpery dole is the one who is willing to take the trumpery wage, and between the two insufficiencies lives in constant misery. If the dole were withheld she would have to hold out for something higher, something more strictly a "living wage." She could not be worse off than she is just now and live at all. Who, then, shall we say, gets the benefit of the parish charity? Not really the worker, who, as we have said, could not exist on less, wherever she got it from. The person who really profits is the employer, who gets his labour under cost price, cost price of labour being such amount of food, clothing, and shelter as serves to keep the worker in life and in some measure of efficiency. Or if, forced by the competition of other employers, he sells his goods at a rate which would be impossible if he paid a better wage to his workers, then the benefit goes a step further on, to the purchaser who buys the cheap (and not infrequently nasty) things thus made.

It should hardly be necessary at this time of day to point out that this always has been and always will be the result of such charities as become grants in aid of wages. The experience of the old Poor Law Act should have been sufficient. But it can hardly be too frequently repeated to those that if they cannot give such charity as will permanently raise the recipients above the need of alms, or else completely maintain them, they had better give nothing at all. Insufficient doles do those who get them little good, and indirectly, as we have tried to show, do those workers who do not get them an infinite deal of harm.

